# Urinary Microbiome and Psychological Factors in Women with Overactive Bladder

**DOI:** 10.3389/fcimb.2017.00488

**Published:** 2017-11-27

**Authors:** Peng Wu, Yang Chen, Jie Zhao, Guihao Zhang, Jiawei Chen, Junpeng Wang, Huijian Zhang

**Affiliations:** ^1^Department of Urology, Nanfang Hospital, Southern Medical University, Guangzhou, China; ^2^School of Pharmaceutical Sciences, Southern Medical University, Guangzhou, China

**Keywords:** bacteria, depression, overactive bladder, psychology, urinary microbiome

## Abstract

**Objectives:** Emerging evidence indicates that alterations to the urinary microbiome are related to lower urinary tract symptoms. Overactive bladder (OAB) is a common disorder with complex etiologies and usually accompanied by psychological diseases. More information concerning the urinary microbiome and psychological factors in OAB is required. The aim of this study was to characterize the female urinary microbiome associated with OAB and investigate the relationships between urinary microbiome and psychological factors.

**Methods:** Thirty women with OAB and 25 asymptomatic controls were recruited and asked to finish the Overactive Bladder Symptom Score, Self-Rating Anxiety Scale and Self-Rating Depression Scale. Urine specimens were collected by transurethral catheterization and processed for 16S rRNA gene sequencing using Illumina MiSeq. Sequencing reads were processed using QIIME. LEfSe revealed significant differences in bacterial genera between controls and OAB patients. The relationships between the diversity of the urinary microbiome and psychological scores were identified by Pearson's correlation coefficient.

**Results:** We found that bacterial diversity (Simpson index) and richness (Chao1) were lower in OAB samples compared to controls (*P* both = 0.038). OAB and control bacterial communities were significantly different (based on weighted UniFrac distance metric, *R* = 0.064, *P* = 0.037). LEfSe demonstrated that 7 genera were increased (e.g., *Proteus* and *Aerococcus*) and 13 were reduced (e.g., *Lactobacillus* and *Prevotella*) in OAB group compared to controls. There were negative correlations between scores on Self-Rating Depression Scale and both richness (Chao1, *r* = −0.458, *P* = 0.011) and diversity (Shannon index, *r* = −0.516, *P* = 0.003) of urinary microbiome in OAB group. Some bacterial genera of OAB women with anxiety or depression were significantly different from those without.

**Conclusions:** The aberrant urinary microbiome with decreased diversity and richness may have strong implications in pathogenesis and treatment of OAB. Psychological conditions were correlated with characteristics of urinary microbiome in women with OAB. Further research is needed to understand the connection between central nervous system and urinary microbiome.

## Introduction

The microbiome is increasingly considered as an essential factor in human health and disease (Young, 2017). Following the completion of the National Institutes of Health Human Microbiome Project, there have been numerous studies identifying and cataloging the microbiome in physiological and pathological states including gastrointestinal diseases, metabolic diseases, infectious diseases, and cancer (Whiteside et al., 2015). The female urinary tract is non-sterile, as bacteria were detected in urines from asymptomatic people using 16S rRNA gene sequence analysis and/or an expanded quantitative urine culture (EQUC) (Fouts et al., 2012; Wolfe et al., 2012; Hilt et al., 2014; Pearce et al., 2014). Additionally, studies have shown that standard culture missed 90% of the bacteria detected by EQUC (Hilt et al., 2014; Pearce et al., 2014). Similar to the influence of microbial communities in other niches, alterations to the urinary microbiome may have an effect on urinary tract disorders. Emerging evidence indicates that shifts in the normal microbiome of the bladder may play an important role in pathophysiology of lower urinary tract symptoms (LUTS) (Siddiqui et al., 2012; Pearce et al., 2014; Nickel et al., 2015; Karstens et al., 2016; Abernethy et al., 2017; Thomas-White et al., 2017).

Overactive bladder (OAB) is a subset of LUTS characterized by urgency, with or without urgency urinary incontinence (UUI), usually with frequency and nocturia, in the absence of urinary tract infection (UTI), or other identifiable causes (Abrams et al., 2002). OAB symptoms affect social, psychological, occupational, domestic, physical, and sexual aspects of life and lead to a heavy cost (Stewart et al., 2003). Some OAB symptoms are clearly due to neuromuscular issues and muscarinic receptors, but some individuals with OAB do not respond to anti-muscarinic receptor, botox, or other treatments (Gormley et al., 2015), which suggests that their symptoms might arise from other causes or that the causes are complex. Therefore, it is an urgent need to further investigate the pathogenesis of OAB and identify new therapeutic target.

A brain–gut–microbiome axis between the brain, the gut, and the gut microbiome has been well identified (Mayer et al., 2014). The urinary microbiome might play similar roles, given the well-known connection between central nervous system and bladder function (Tadic et al., 2010). OAB patients had higher levels of depression, anxiety and embarrassment than asymptomatic people (Kinsey et al., 2016). Bradley et al. found that female veterans with anxiety symptoms were more likely to have bothersome urgency incontinence and/or frequency symptoms (Bradley et al., 2014). It was reported that OAB patients with depression had more severe urinary incontinence symptoms, greater bother and more impact on quality of life compared to those without depression (Lai et al., 2016). There have been some studies on the urinary microbiome of OAB/UUI (Hilt et al., 2014; Pearce et al., 2014; Karstens et al., 2016; Curtiss et al., 2017; Thomas-White et al., 2017), but no previous study researched the urinary microbiome considering psychological disorders. Our primary purpose was to characterize the female urinary microbiome associated with OAB in China and investigate relationships between the microbiome and psychological factors.

## Materials and methods

### Subject recruitment and urine collection

Between September 2016 and March 2017, adult patients aged 18 or above, diagnosed with OAB, and asymptomatic controls were recruited into this study at Nanfang Hospital in China. Patients must complain of urinary urgency, with or without urgency incontinence, usually with frequency and nocturia, in the absence of infection or other identifiable causes, in accordance with the 2002 ICS definition of OAB (Abrams et al., 2002). Asymptomatic controls must have no prior diagnosis of OAB or interstitial cystitis/bladder pain syndrome, no pelvic pain, and no evidence of urinary infection. Subjects with a history of urethral stricture disease, urinary retention, pelvic radiation, tuberculosis cystitis, cyclophosphamide cystitis, genitourinary cancer, urinary stones, neurogenic bladder, a documented positive urine culture in the past 6 weeks, or a post-void residual volume ≥150 mL were not eligible. The Medicine Institutional Review Board of Southern Medical University approved this study, and written informed consents from all participants were obtained. All participants were required to finish Overactive Bladder Symptom Score (OABSS), Self-Rating Anxiety Scale (SAS, standard score ≥ 50 indicates anxiety) and Self-Rating Depression Scale (SDS, standard score ≥ 53 indicates depression). Many studies investigated the urinary microbiome based on the collection of mid-stream urine into a sterile container (Siddiqui et al., 2012; Lewis et al., 2013; Thomas-White et al., 2017). However, the collection of mid-stream urine cannot avoid contamination from the distal urethra, vulva, or vagina. Sterile alternatives like transurethral catheter and suprapubic aspirate might be more adequate and elaborate for scientific exploration of the bladder microbiome (Wolfe et al., 2012; Hiergeist and Gessner, 2017). Therefore, 50 ml of urine from each participant was collected through transurethral catheterization in this study. Twenty milliliter of the specimen was tested by standard cultivation to exclude UTI. The rest of specimen (30 ml) was kept at 4°C, and immediately shifted to laboratory within an hour for centrifugation at 16,000g for 10 min. The pellets were stored at −80°C until further processing.

### DNA isolation and 16S rRNA gene sequencing

DNA extraction was performed using the cultured cells protocol supplied with the DNeasy Blood and Tissue Kit (Qiagen, Germany) in a laminar flow hood to avoid contamination. The concentration of extracted DNA was determined using a Nanodrop ND-1000 spectrophotometer (Thermo Electron Corporation, USA). PCR amplification of 16S rDNA sequences was performed using primer sets specific for V4 regions. Extraction negative controls (no urine) and PCR negative controls (no template) were included to assess contribution of extraneous DNA from reagents. Final PCR products were purified from unincorporated nucleotides and primers using the Qiaquick PCR purification kit (Qiagen, Valencia, USA). Purified samples were normalized to equal DNA concentration and sequenced using the Illumina Miseq sequencer (Illumina, USA). The 16S rRNA gene sequences have been submitted to the Short Read Archive (SRA) under accession number SUB2843807.

### Statistical analysis

#### Clinical data analysis

Differences in demographic characteristics between patients and controls were evaluated using Mann-Whitney *U*-test for continuous variables and Pearson's chi-square test for count data. Bivariate correlation analyses were conducted to detect the direction and strength of relations between clinical data and indices of bacterial alpha diversity using Pearson's correlation. Statistical analysis was performed using the Statistical Package for Social Science (SPSS, version 21, USA). For differentially abundant taxa (only taxa with mean relative abundance more than 1% were tested) between cohorts, Wilcoxon rank sum test was applied, and Benjamini-Hochberg false discovery rate correction was performed in R (version 3.4.1, stats package). Statistical tests were based on two-tailed probability. The conventional *P* < 0.05 was used to assess the statistical significance.

#### Bioinformatics analysis

The wrapper package Quantitative Insights Into Microbial Ecology (QIIME) was applied to process the raw reads to create an operational taxonomic units (OTUs) table (Caporaso et al., 2010). Sequences were clustered into individual OTUs at a default similarity level of 97% using an open reference picking strategy with Uclust, and subsequently, chimera detection was performed using the UCHIME method (Edgar, 2010). A single representative sequence from each clustered OTU was used to align to the Greengenes database using Ribosomal Database Project Classifier (Wang et al., 2007).

Alpha diversity was evaluated by calculating four indices using QIIME, including the Observed species, Chao1, Shannon, and Simpson indices. Pielou index is an indicator of species evenness generated in R (version 3.4.1, vegan package), whereas Chao1 and Observed species represent bacterial richness. Shannon and Simpson indices are quantitative measures of bacterial diversity reflecting both species richness and evenness. The difference of alpha diversity was evaluated by Kruskal–Wallis test. To compare microbial composition between samples, beta-diversity was measured by calculating the Bray Curtis, weighted UniFrac and unweighted UniFrac distances. Principal coordinate analysis (PCoA) was applied on the distance matrices to generate three-dimensional plots in QIIME. The analysis of similarities (ANOSIM) of variance of Bray Curtis, weighted UniFrac, and unweighted UniFrac distances within R software was used to calculate *P*-values and test for significant differences in beta-diversity between groups.

To identify significantly different bacteria as biomarkers between groups at genus level, taxa summaries were reformatted and inputted into Linear discriminant analysis effect size (LEfSe) via the Huttenhower Lab Galaxy Server (Segata et al., 2011). In the settings of LEfSe, the Kruskal-Wallis sum-rank test (α = 0.05) was used to detect taxa with significant differential abundance at first; as a second step, biological consistency was then investigated with a set of pairwise tests among subclasses using the Wilcoxon rank-sum test; finally, linear discriminant analysis (LDA) was used to estimate the effect size of differentially abundant genera (Segata et al., 2011). The threshold on the logarithmic LDA score for discriminative features was 2.5.

## Results

### Participant demographic characteristics and clinical symptoms

Thirty women with OAB and 30 asymptomatic controls were recruited. However, five samples in the control group were excluded because three of them contained a few sequencing reads and two were suspected to contain contaminants. There was no significant difference in the demographic characteristics between two cohorts (Table 1, Supplementary Table 1), except the previous anticholinergic use (OAB 37%, control 0%, *P* = 0.001). The median age of patients was 27.5 years and of controls was 26.0 years (*P* = 0.258). Most of these participants were currently married (OAB 70%, control 75%) and premenopausal (OAB 87%, control 80%). For the OABSS, the total score and some sub-scores (daytime frequency, nighttime frequency and urgency) were significantly higher in the OAB group than in the controls (Table 1, *P* all < 0.001). Twelve (40%) of the OAB group had anxiety (median score 48.0), one of which reported severe anxiety, and 14 (47%) suffered from mild or moderate depression (median score 48.5). In the control group, 3 (12%) had mild or moderate anxiety (median score 41.0), and 5 (20%) suffered from mild or moderate depression (median score 40.0). The prevalence of anxiety and the prevalence of depression in the OAB group were both markedly higher than in the control group (*P* < 0.05).

**Table 1:** Comparisons of demographic characteristics and scale scores between OAB patients and asymptomatic controls.

**Characteristic**	**Patients with OAB (*n* = 30)**	**Asymptomatic controls (*n* = 25)**	***P*-value**
Age (y)	27.5 (26.0, 35.3)	26.0 (23.0, 47.5)	0.258
BMI	20.6 (18.6, 22.2)	20.0 (19.0, 21.8)	0.754
Currently married	21 (70)	19 (75)	0.619
Ever pregnant	19 (63)	14 (56)	0.580
Premenopausal	26 (87)	20 (80)	0.506
Estrogen treatment	3(10)	0	0.104
DM	2 (6)	0	0.188
HP	1 (3)	0	0.357
Previous anticholinergic use	11 (37)	0	0.001
Gynecologic diseases	3 (10)	5 (20)	0.295
Pelvic surgery	2 (6)	1 (4)	0.665
OABSS	8.0 (6.0, 10.0)	1.0 (0, 2.5)	<0.001
Q1. Daytime frequency	2.0 (1.0, 2.0)	0 (0, 1.0)	<0.001
Q2. Nighttime frequency	2.0 (2.0, 3.0)	0 (0, 1.0)	<0.001
Q3. Urgency	4.0 (2.8, 5.0)	0 (0, 1.0)	<0.001
Q4. Urgency incontinence	0 (0, 0)	0 (0, 0)	0.572
SDS	48.5 (45.0, 58.8)	40.0 (30.5, 48.5)	<0.001
Mild or moderate depression	14 (47)	5(20)	0.038
Severe depression	0	0	ns
SAS	48.0 (46.0, 59.3)	41.0 (33.0, 45.5)	<0.001
Mild or moderate anxiety	11 (37)	3 (12)	0.037
Severe anxiety	1 (3)	0	0.357

*Data are reported as median (interquartile range) for continuous variables or n (%) for counts. BMI, Body mass index; DM, diabetes mellitus; HP, hypertension; OABSS, Overactive Bladder Symptom Score; SDS, Self-Rating Depression Scale; SAS, Self-Rating Anxiety Scale; and ns, not significant (based on P < 0.05). Standard scores on SAS ≥ 50 indicate the presence of anxiety; standard scores on SDS ≥ 53 indicate depression*.

### Sequencing data, alpha, and beta diversity

We obtained 6,605,388 sequences from the 55 samples. The median number of reads in OAB group was 110,250, and in the control group was 102,125 (Table 2, *P* = 0.685). The reads were classified into 39,905 OTUs that were used for downstream analyses. No difference was found in the number of OTUs between the two cohorts (Table 2, *P* = 0.073).

**Table 2:** Comparisons of parameters of microbiome and mean relative abundances of bacteria (as percent reported by phyla: family) in OAB urine and control urine.

	**Patients with OAB (*n* = 30)**	**Asymptomatic controls (*n* = 25)**	***P*-value**
**PARAMETER**			
Number of reads	110,250 (64,210, 151,181)	102,125 (76,102, 153,111)	0.685
Number of OTUs	2,203 (1,550, 4,934)	1,979 (1,015, 2,817)	0.073
Observed species	759 (482, 1,099)	915 (644, 1,714)	0.088
Chao1	1,622.7 (997.2, 2615.9)	2,092.8 (1606.4, 3736.3)	0.038
Shannon index	4.7 (3.5, 5.2)	4.7 (3.7, 6.4)	0.243
Simpson index	0.8 (0.7, 0.9)	0.9 (0.8, 10.0)	0.038
Pielou index	0.3 (0.2, 0.5)	0.4 (0.3, 0.6)	0.205
**RELATIVE ABUNDANCE (%)**			
*Firmicutes*	42.7	56.4	0.152
*Lactobacillaceae*	30.2	41.2	0.945
*Veillonellaceae*	5.4	5.2	0.658
*Tissierellaceae*	2.3	4.7	0.227
*Proteobacteria*	25.0	14.3	0.960
*Enterobacteriaceae*	17.6	8.1	0.099
*Actinobacteria*	19.8	6.8	0.048
*Bifidobacteriales*	14.8	3.6	0.012
*Bacteroidetes*	8.1	14.3	0.115
*Prevotellaceae*	6.2	13.5	0.226
*Fusobacteria*	2.2	1.8	0.822
*Synergistetes*	<1	1.4	0.012

*Data are reported as median (interquartile range) for parameters. OAB, Overactive bladder; OTUs, operational taxonomic units*.

**Figure 1:**
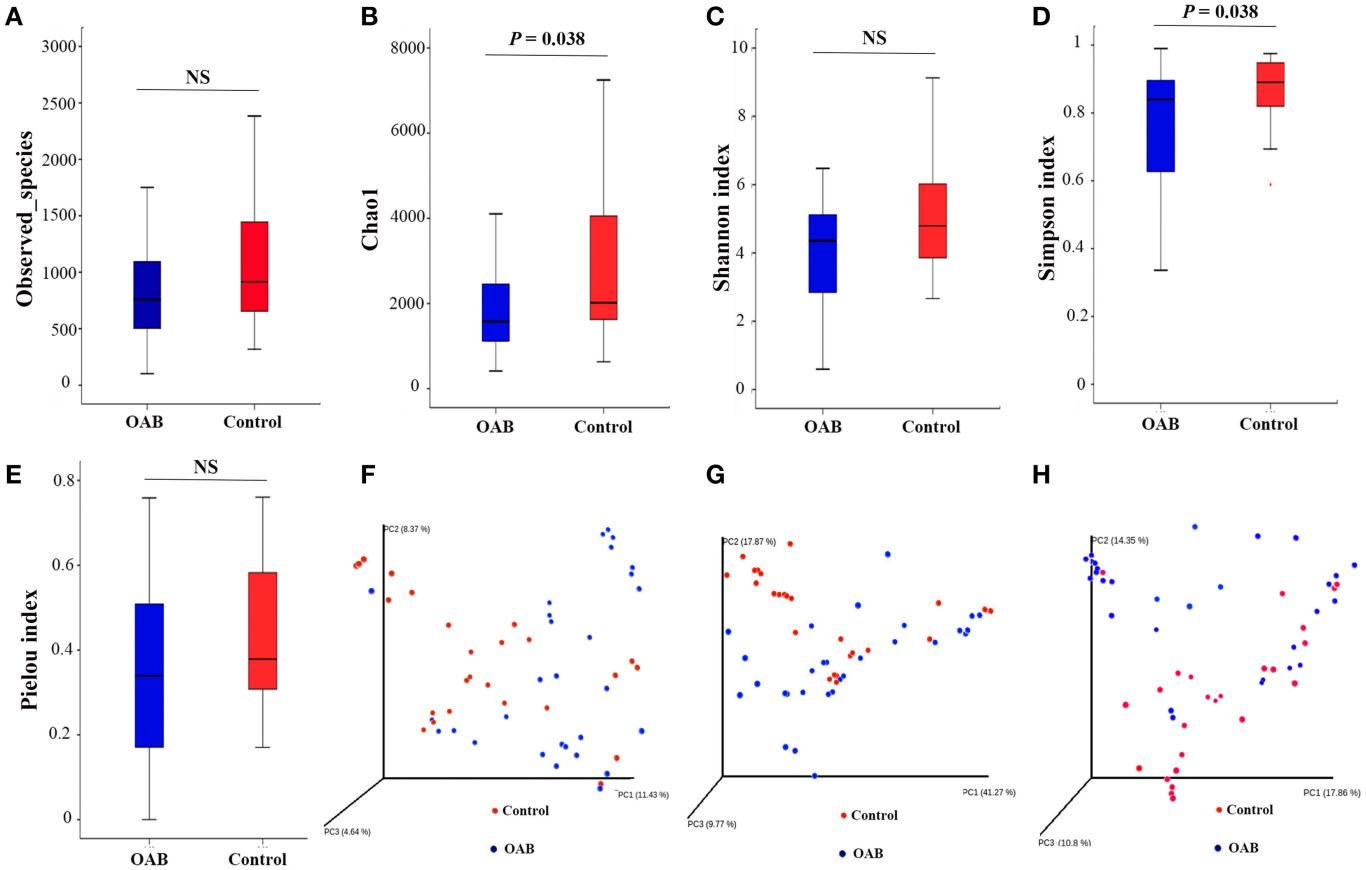
Alpha diversity and principal coordinate analysis for control and OAB urinary microbiomes. Observed species **(A)**; Chao1 **(B)**; Shannon index **(C)**; Simpson index **(D)**; Pielou index **(E)**. Principal coordinate analysis plot of the urinary microbiome based on the unweighted **(F)** or weighted **(G)** UniFrac and Bray-Curtis **(H)** distance metrics.

The indices of bacterial alpha diversity were listed in Table 2 and Supplementary Table 2. There were no differences in the Observed species (Figure 1A) and Shannon index (Figure 1C) between OAB and controls. The Chao1 was significantly lower in the OAB cohort than in the control cohort (*P* = 0.038; Figure 1B). The Simpson index illustrated the bacterial diversity was markedly lower in the OAB patients (*P* = 0.038; Figure 1D). However, the Pielou index indicated that there was no significant difference in bacterial evenness between the two groups (*P* = 0.205; Figure 1E).

**Figure 2:**
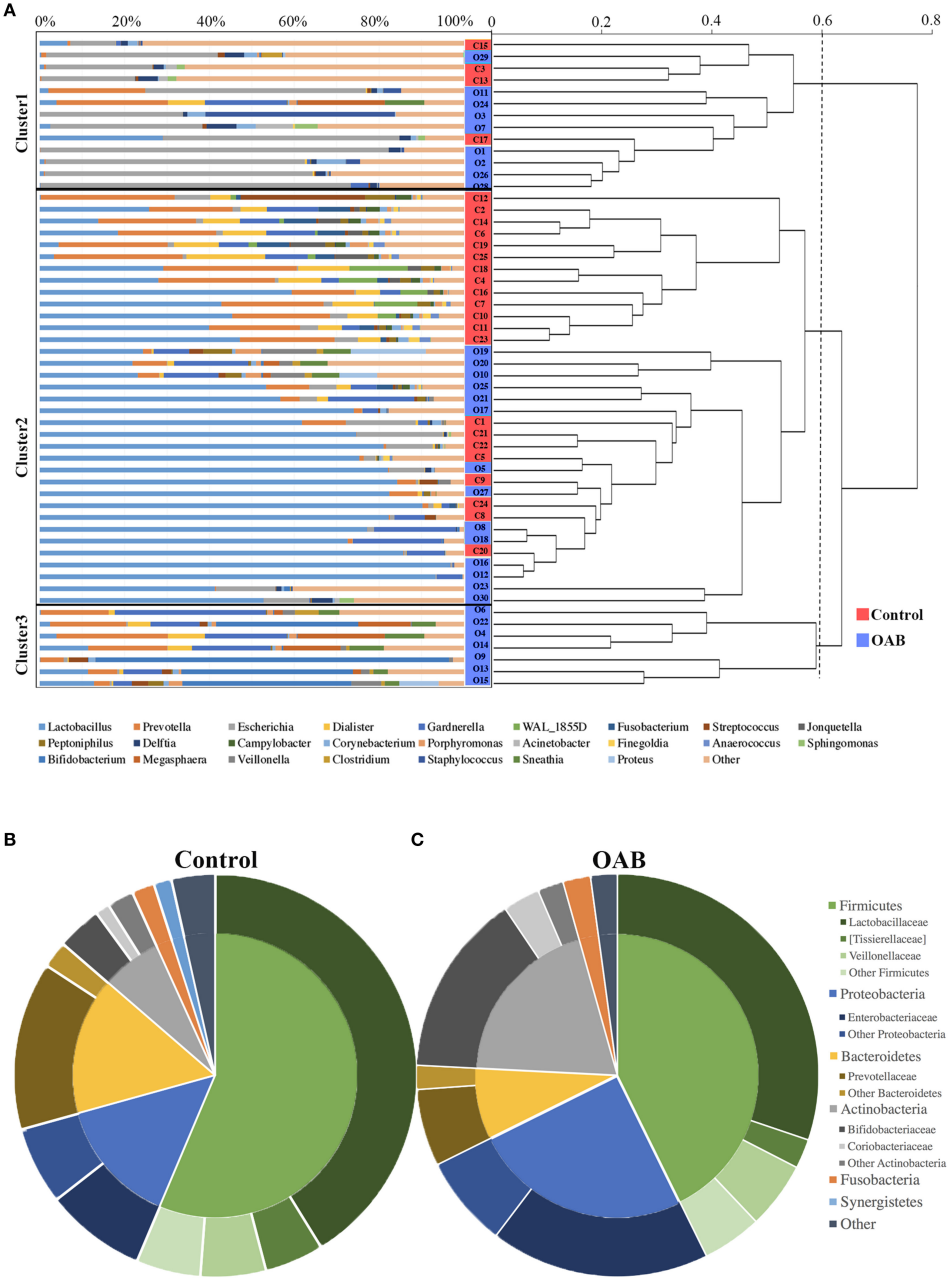
The urinary microbiome profile of participants. The urinary microbiome profiles of the two cohorts cluster together, as showed in the dendrogram (right; based on the weighted UniFrac distance metric) and by the dominant genera present, as depicted in the histogram (left) **(A)**. Comparison of taxonomic assignments for the controls **(B)** and OAB samples **(C)** at the Phyla level (inner circle) and family level (outer circle). Bacterial genera with a relative abundance <0.5%, are grouped as “Other.”

The PCoA was performed to measure the extent of the similarity of microbial communities in the two cohorts based on unweighted UniFrac (Figure 1F), weighted UniFrac (Figure 1G) and Bray Curtis (Figure 1H) distance metrics. Some OAB patients clustered away from controls in the PCoA plot and hierarchical clustering based on weighted UniFrac distance metric (Figures 1G, 2A), but there were overlapping regions where other OAB women and controls were located. Mann-Whitney *U*-test was applied to analyze variables between OAB patients clustered away from controls and other patients mixed with controls. We found that scores on OABSS, SAS, and SDS in OAB patients clustered away from controls (cluster 1 and cluster 3 in Figure 2A) were higher than in patients mixed with controls (cluster 2 in Figure 2A), although without statistical significance (Supplementary Table 3). Specifically, ANOSIM revealed that there were significant differences in bacterial communities between OAB and control groups using the Bray-Curtis distance metric (statistic *R* = 0.055, *P* = 0.038, number of permutations = 999) and weighted UniFrac distance metric (statistic *R* = 0.064, *P* = 0.037, number of permutations = 999). However, this clustering was not significant when the analysis was based on unweighted UniFrac distance metric (statistic *R* = 0.023, *P* = 0.068, number of permutations = 999).

### Relative abundances of urinary bacteria in control and OAB samples

**Figure 3:**
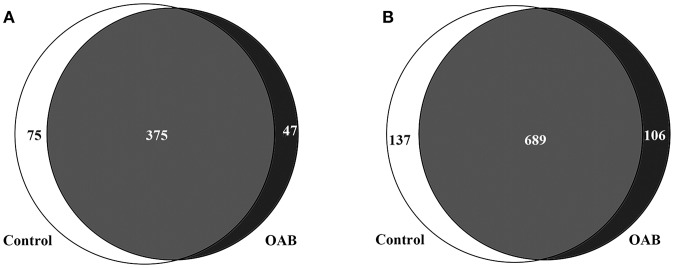
Venn diagrams depicting the number of bacterial families **(A)** and genera **(B)** that are shared and unique between control and OAB urine.

The genera compositions of all samples were also demonstrated in Figure 2A and Supplementary Table 4. The control group had a higher degree of diversity than the OAB group. Half of control samples were dominated by *Lactobacillus*. However, the microbiome of OAB group displayed greater variability, and could be divided into three clusters. The mean relative abundances of various bacterial phyla and families in each group were listed in Table 2 and summarized in Figures 2B,C. The most frequently detected phylum was *Firmicutes* (42.7% OAB, 56.4% control), followed by *Proteobacteria* (25.0% OAB, 14.3% control), *Actinobacteria* (19.8% OAB, 6.8% control, *P* = 0.048) and *Bacteroidetes* (8.1% OAB, 14.3% control). At the family level, *Bifidobacteriaceae* (*P* = 0.012) was significantly more abundant in the OAB group. Venn diagrams demonstrated that 375 families (Figure 3A) and 689 genera (Figure 3B) were shared between the two cohorts. The bacterial compositions of all samples at different levels were shown in Supplementary Figure 1.

### Specific genera associated with OAB

**Figure 4:**
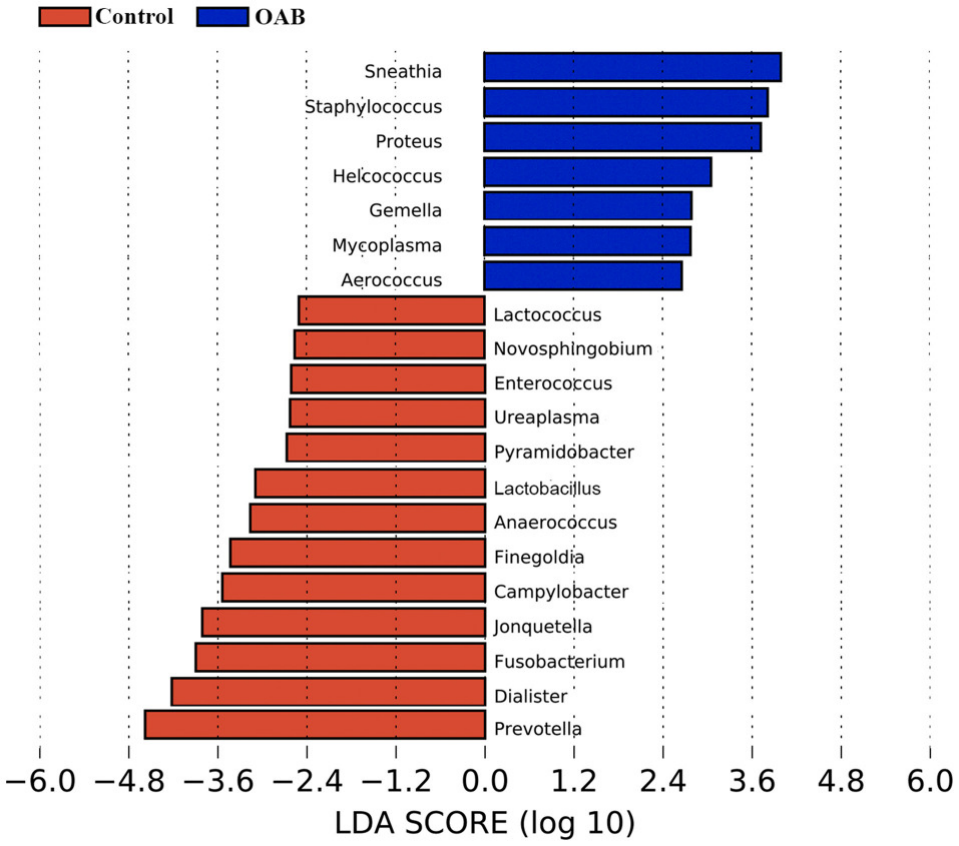
LEfSe analyses of urinary microbiomes of OAB patients compared with controls. Genera enriched for controls in red; OAB patients enriched genera in blue. Only genera meeting a linear discriminant analysis score threshold >2.5 are shown.

The LEfSe algorithm was utilized to identify the specific bacteria genera associated with OAB (Figure 4). By specifying control and OAB as distinct classes, the LEfSe revealed that 13 genera were underrepresented in the OAB group, including *Prevotella, Dialister, Fusobacterium, Jonquetella, Campylobacter, Finegoldia, Anaerococcus, Lactobacillu, Pyramidobacter, Ureaplasma, Enterococcus, Novosphingobium*, and *Lactococcus*. In contrast, there were seven genera overrepresented in OAB group, including *Sneathia, Staphylococcus, Proteus, Helcococcus, Gemella, Mycoplasma*, and *Aerococcus*.

### Urinary microbiome and psychological symptoms in OAB patients

**Figure 5:**
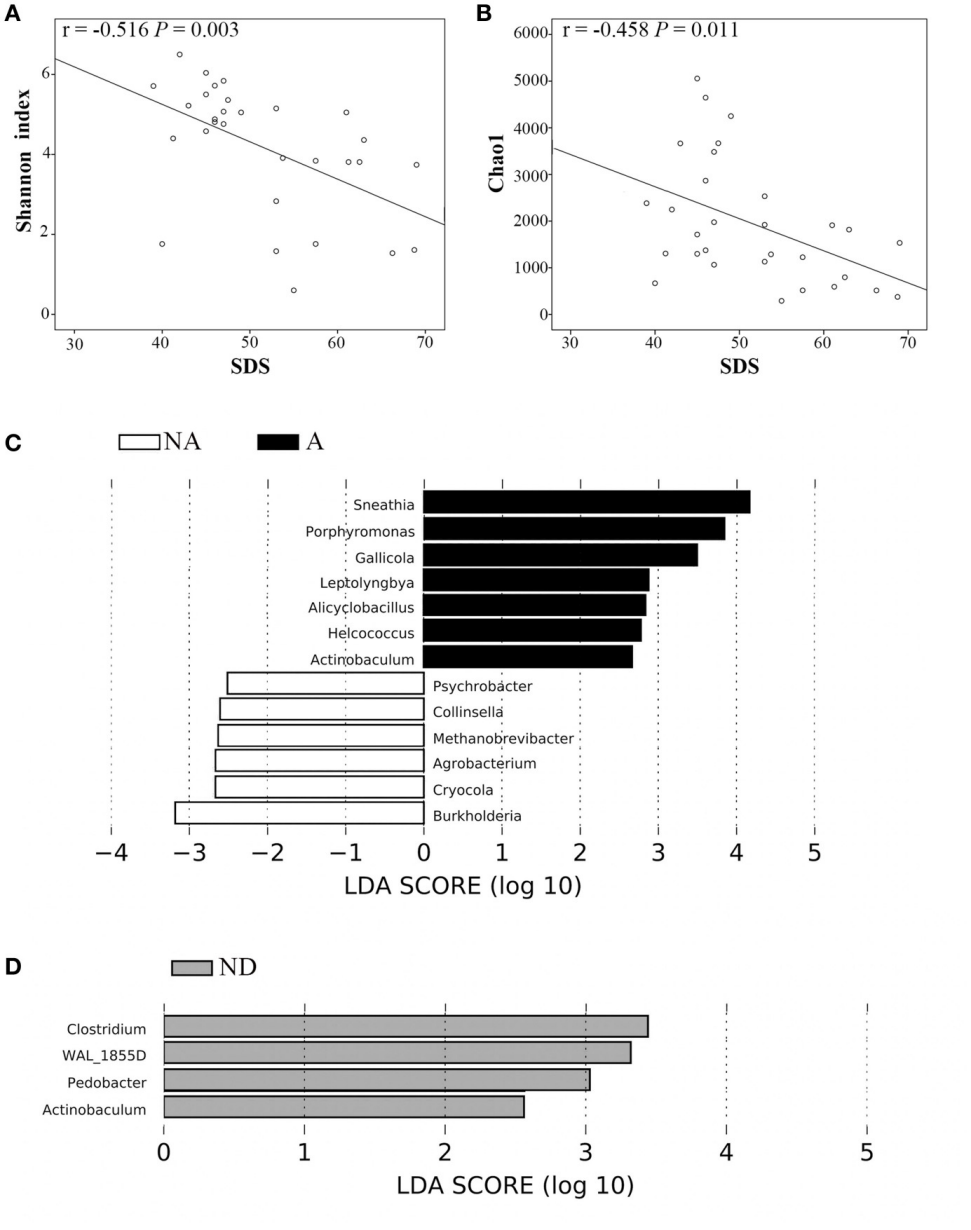
Correlations between variables and significantly different bacterial genera between OAB patients with and without anxiety or depression. Higher SDS score inversely correlated with both Shannon index **(A)** and Chao1 **(B)**. Bacterial genera differed significantly between OAB patients with and without anxiety based on LEfSe analyses **(C)**. Bacterial genera differed significantly between OAB patients with and without depression based on LEfSe analyses **(D)**. Only genera meeting a linear discriminant analysis score threshold >2.5 are shown. A, Anxiety; NA, non-anxiety; ND, non-depression; SDS, Self-rating Depression Scale.

We compared several variables between anxiety group and non-anxiety group, and depression group and non-depression group in OAB patients, respectively. We found that OAB patients with anxiety or depression reported significantly higher scores on OABSS (Table 3, *P* all <0.05). The alpha diversity indices (Observed species, Chao1, Simpson, and Shannon indices) were markedly lower in depression cases than in non-depression cases (Table 3, *P* all <0.01). Higher score on SDS inversely correlated with both Shannon index (*r* = −0.516, *P* = 0.003, Figure 5A) and Chao1 (*r* = −0.458, *P* = 0.011, Figure 5B). LEfSe algorithm was also applied to identify the specific genera that were differentially represented in different psychological status in OAB patients. Based on LEfSe resluts, *Burkholderia, Cryocola, Agrobacterium, Methanobrevibacter, Collinsella*, and *Psychrobacter* were relatively more abundant in the non-anxiety cohort compared to the anxiety cohort (Figure 5C). In contrast, *Sneathia, Porphyromonas, Gallicola, Leptolyngbya, Alicyclobacillus, Helcococcus*, and *Actinobaculum* were significantly enriched in the OAB patients with anxiety (Figure 5C). Compared to the depression cohort, more *Actinobaculum, Pedobacter, WAL_1855D*, and *Clostridium* were found in the non-depression cohort (Figure 5D).

**Table 3:** Comparisons of clinical symptoms and biodiversity of urinary microbiome between OAB patients with and without psychological disorders.

	**SAS**		***P*-value**	**SDS**		***P*-value**
	**<50 (*n* = 18)**	**≥50 (*n* = 12)**		**<53 (*n* = 16)**	**≥53 (*n* = 14)**	
SAS	45.6 (43.0, 47.3)	60.6 (55.8, 63.8)	<0.001	50.6 (43.8, 60.5)	51.3 (46.0, 58.5)	0.697
SDS	48 (41, 55)	51 (46, 62)	0.305	45.0 (42.0, 47.0)	58.5 (52.5, 63.0)	<0.001
OABSS	6.9 (5.0, 8.3)	9.1 (8.0,10.0)	0.007	7.0 (6.0, 8.0)	8.6 (7.5, 10.0)	0.043
Q1. Daytime frequency	1.4 (1.0, 2.0)	2.0 (2.0, 2.0)	0.010	1.6 (1.0, 2.0)	1.8 (1.8, 2.0)	0.313
Q2. Nighttime frequency	2.0 (1.0, 2.0)	2.3 (2.0, 3.0)	0.285	2.1 (2.0, 2.8)	2.2 (1.8, 3.0)	0.580
Q3. Urgency	3.2 (0, 0)	4.3 (3.3, 5.0)	0.048	3.2 (2.0, 4.0)	4.1 (4.0, 5.0)	0.034
Q4. Urgency incontinence	0.3 (0, 0)	0.5 (0, 0)	0.951	0.2 (0, 0)	0.7 (0, 1.0)	0.334
Observed species	826.5 (384.0, 1136.0)	785.0 (515.5, 1052.0)	0.987	1,065.1 (782.8, 1297.3)	518.3 (307.5, 679.8)	0.002
Chao1	1,836.4 (1014.3, 2420.5)	2,087.6 (862.8, 3465.8)	0.723	2,604.3 (1322.5, 3668.5)	1,174.1 (514.8, 1840.6)	<0.001
Shannon index	4.0 (2.7, 5.4)	4.5 (3.8, 5.2)	0.662	5.1 (4.8, 5.7)	3.1 (1.6, 4.0)	<0.001
Simpson index	0.7 (0.4, 0.9)	0.8 (0.8, 0.9)	0.146	0.8 (0.8, 0.9)	0.6 (0.4, 0.9)	0.002
Pielou index	0.3 (0.2, 0.4)	0.5 (0.3, 0.6)	0.217	0.3 (0.2, 0.5)	0.4 (0.2, 0.6)	0.423

*Data are reported as median (interquartile range). OABSS, Overactive Bladder Symptom Score; SDS, Self-Rating Depression Scale; SAS, Self-Rating Anxiety Scale*.

## Discussion

In the present study, we characterized the urinary microbiomes of female OAB patients and asymptomatic women using high throughput sequencing of the bacterial 16S rRNA gene. The bacterial diversity and richness were decreased in the OAB urinary microbiome. Furthermore, 7 genera were increased and 13 were reduced compared to the control samples. We also found negative correlations between the depression score and both bacterial richness and diversity in the OAB patients, which inferred that psychological factors might play an important role in the urinary microbiome.

There have been some studies on the urinary microbiome of OAB/UUI. Hilt et al. identified bacteria associated with OAB by EQUC, and found that *Aerococcus* and *Actinobaculum* were isolated only from OAB urine (Hilt et al., 2014). Another study analyzing urinary microbiome collected from women with UUI and asymptomatic controls found that the microbiome from UUI displayed decreased *Lactobacillus* and increased *Gardnerella* abundance by sequence analysis, and nine genera were more frequently cultured from the UUI cohort by culture techniques (Pearce et al., 2014). Karstens et al. identified statistically significant differences in the relative abundance of specific bacteria in urine from women with UUI compared to controls using high throughput sequencing (Karstens et al., 2016). They also found that lower microbial diversity was associated with increased symptom severity in women with UUI. Our study shared some results with the above studies, such as increased genera (e.g., *Aerococcus* and *Staphylococcus*) and decreased genera (e.g., *Prevotella* and *Lactobacillus*) in the patient group. Hilt's group has evaluated the urinary microbiomes of hundreds of women with OAB/UUI and a smaller but substantial number of asymptomatic controls (Hilt et al., 2014; Pearce et al., 2014). It seems pretty clear that there are some bacterial species (including *Aerococcus*) associated with urinary symptoms and not with asymptomatic controls. However, there were several differences between these studies. An obvious difference was that *Gardnerella*, a genus representing one of the major urotypes in above previous studies, had low relative abundance in both cohorts in our study, similar to another study from China (Liu et al., 2017). Differences could be partly due to different clinical characteristics. Previous studies included populations with more severe urinary symptoms than our study. In addition, differences in reference databases used which can cause differences in the types of bacteria identified might also contribute to the discrepancies. Our study used the GreeneGenes database for taxanomic assignment, whereas other studies used the SILVA database. Another reason might be that the ethnic group in our study was different from that in the previous studies above. Notably, the gut microbiome was compositionally affected by geographical and ethnic factors (Prideaux et al., 2013). Future study focusing on whether there are differences in the urinary microbiomes between different racial types is needed.

The Chao1 and Simpson index showed that the bacterial richness and diversity were lower in OAB samples compared to the asymptomatic samples. However, the bacterial evenness was not significantly different between the two groups, indicating that the shift in diversity was caused by the reduced richness. The urinary microbiome of asymptomatic women was found to be more diverse, and richer in *Lactobacillus* compared to the OAB group. A more diverse and *Lactobacillus*-dominated urinary microbiome might play a potentially protective role, which is consistent with arguments of other studies (Brubaker et al., 2014; Pearce et al., 2014). The profile of urinary microbiome in interstitial cystitis was also found to be less diverse and less likely to contain *Lactobacillus* species (Abernethy et al., 2017). This suggests that the intriguing possibility that specific microbial patterns may be linked to specific symptoms, regardless of diagnosis, which provides a great enlightenment for future researches of the urinary microbiome and the algorithms for the diagnosis and treatment of LUTS.

Although, we detected differences in several biologically plausible variables between the two groups, there was a great deal of individual variability among the OAB patients. As determined by PCoA, there are overlapping regions where a subgroup of samples from OAB and controls are located. It suggests that some samples of OAB patients are very similar to controls, but some urinary microbiomes from OAB patients are quite distinct. Although, it was not statistically significant, the OAB patients whose urinary microbiomes were obviously different from the controls reported higher scores on OABSS, SAS, and SDS than in patients similar to controls. Moreover, different psychological conditions were associated with different severities of symptom and characteristics of urinary microbiome (Table 3). Similar to UUI (Pearce et al., 2014), this could be due to internal heterogeneity in the OAB population. The status of the urinary microbiome and mental factors should all be taken into consideration for clinical phenotyping of patients. The careful phenotyping of patients gives a better understanding of heterogeneous pathology, and provides the basis for precise and personalized medicine, such as targeted therapy through removal of specific pathogens and the restoration of normal microbiome supplemented by psychotherapy.

Pathogenic organisms invading in urothelial cells were confirmed to be able to persist for a long time and serve as the primary source for bacterial expansion (Hunstad and Justice, 2010). Among the urine samples collected from chronic LUTS patients, 75% showed evidence of infected urothelial cells, which might stimulate the apoptotic process in urothelial cells and increase exposure of the underlying mucosal layer to irritative stimuli (Horsley et al., 2013). Low-count bacteriuria and intracellular bacterial communities were more prevalent among OAB women when a lower cut-off threshold (10^2^ CFU/mL) was used (Khasriya et al., 2010). We identified 20 bacteria with significantly differential relative abundances between OAB microbiome and control microbiome. Of the 7 bacteria that had increased relative abundance in OAB urinary microbiome, some (*Proteus, Staphylococcus*, and *Mycoplasma*) are commonly seen in patients with UTI. This suggests that a persistent low grade infection by bacteria that are not commonly detected by standard culture could potentially be responsible for the OAB symptoms. It is a new dogma that the urine is not sterile and that many uropathogens are not detected by the standard culture method. The microbiome might be a new paradigm in urogenital infections and diseases, and influence the future classification of UTI.

There is an increasing understanding of the role of the brain–gut–microbiome axis in gastrointestinal diseases (Mayer et al., 2014). Evidence suggests that the axis is a complex bidirectional communication network between the gut microbiome and host, by which microbiome can modulate brain function, and conversely, chronic psychological stress may compound the degree of dysbiosis (Bailey et al., 2011). The urinary microbiome has similar potential. Interstitial cystitis was considered as a central sensitization syndrome with impaired inhibition of descending pain pathways (Ness et al., 2014). For idiopathic OAB patients, central sensitization may also help explain the pathogenesis (Reynolds et al., 2016). A study disclosed that different sets of bacterial taxa in urinary microbiome were increased in the psychosocial-predominant and neurologic-predominant groups of chronic pelvic pain syndrome (Shoskes et al., 2016). In our study, we identified significant correlations between measurements of depression severity and alpha diversity in women with OAB. The data indicates that OAB patients with depression have further reductions in bacterial diversity and richness. Furthermore, some bacterial genera differed significantly between OAB patients with and without anxiety or depression. It is an important finding that requires further studies to establish whether a brain–bladder–microbiome axis exists. It may become possible to manipulate brain status to regulate the urinary microbiome for purposes of treating urinary diseases, and vice versa, in the future.

This cross sectional study did contain several limitations that should be mentioned. First, we only measured the microbiome at one moment in time, thus it was difficult to determine the cause-and-effect relationship between variables. Second, our study was limited by its small sample size. Further expansion of sample size is warranted to update our analysis and do a better comparison to existing studies.

Taken together, we examined differences in the urinary microbiomes of female OAB patients and asymptomatic controls, and then correlated microbiome with psychological conditions. The aberrant urinary microbiome may have strong implications in pathogenesis and treatment of OAB. Further studies are required to confirm the connection between urinary microbiome and central nervous system.

## Author contributions

PW, YC, and JZ: conception and design; YC, GZ, and JC: acquisition of data; YC, JZ, and GZ: analysis and interpretation of data; PW, YC, and JZ: drafting of the manuscript; PW and HZ: critical revision of the manuscript for important intellectual content; PW, YC, JZ, and JW: statistical analysis; PW: obtaining funding; PW: supervision.

### Conflict of interest statement

The authors declare that the research was conducted in the absence of any commercial or financial relationships that could be construed as a potential conflict of interest.
