# Supplementary material for: Urinary Microbiome and Psychological Factors in Women with Overactive Bladder
Source: Front Cell Infect Microbiol. 2017 Nov 27;7:488. doi: 10.3389/fcimb.2017.00488 (PMC5712163; doi:10.3389/fcimb.2017.00488)
Supplement: Supplementary file 1 [file Table1.PDF]

Supplementary table 1-Participant Demographics

ID	BMI	Age (y)	OABSS	Daytime frequency	Nighttime frequency	Urgency	Urgency incontinence	SDS	SAS	Currently married	Ever pregnant	Premenopausal	Estrogen treatment	Diabetes mellitus	Hypertension	Prior anticholinergic drugs	Gynecologic diseases	Pelvic surgery
OAB1	20.55	24	14	2	2	5	5	69	71	0	0	1	0	0	0	0	0	0
OAB2	21.48	65	7	1	2	4	0	41	43	1	1	0	0	0	0	0	0	0
OAB3	21.11	63	11	2	3	5	1	63	53	1	1	0	0	0	0	1	0	0
OAB4	24.97	50	9	2	2	5	0	58	64	1	1	0	0	0	0	1	0	0
OAB5	22.89	46	10	2	3	5	0	58	58	1	1	0	0	0	0	1	0	0
OAB6	21.23	44	6	1	2	3	0	45	42	1	1	1	0	0	0	0	0	0
OAB7	20.55	44	8	2	2	4	0	61	60	1	1	1	0	0	0	0	0	0
OAB8	21.33	42	10	1	2	5	3	40	46	1	1	1	0	0	0	0	0	0
OAB9	19.20	33	5	2	1	1	1	53	46	1	1	1	1	0	0	1	1	0
OAB10	20.58	33	7	2	2	3	0	46	65	1	1	1	0	0	0	1	0	0
OAB11	20.81	31	8	2	1	5	0	61	46	1	1	1	0	0	0	1	0	0
OAB12	24.22	30	5	1	2	2	0	55	48	1	1	1	0	0	0	1	0	0
OAB13	19.63	30	7	2	3	2	0	47	43	1	1	1	0	0	0	0	0	1
OAB14	22.06	28	6	1	1	4	0	54	46	1	1	1	0	1	0	1	0	0
OAB15	22.86	28	9	2	2	5	0	46	63	1	0	1	0	1	1	0	0	0
OAB16	22.86	28	8	2	2	4	0	49	63	1	1	1	0	0	0	0	1	0
OAB17	17.67	27	9	2	3	4	0	53	59	0	0	1	0	0	0	0	0	0
OAB18	17.22	26	5	1	2	2	0	45	49	1	1	1	0	0	0	1	0	0
OAB19	22.49	26	7	2	1	4	0	42	48	0	0	1	0	0	0	0	0	1
OAB20	18.26	26	9	2	3	4	0	54	46	1	1	1	0	0	0	0	0	0
OAB21	19.63	26	7	1	3	2	0	48	48	0	0	1	0	0	0	0	0	0
OAB22	18.59	26	9	2	3	4	0	66	41	0	0	1	0	0	0	0	0	0
OAB23	20.45	26	6	2	1	3	0	43	53	1	1	1	0	0	0	1	0	0
OAB24	17.22	26	10	2	3	5	2	63	47	0	0	1	0	0	0	0	0	0
OAB25	18.07	26	8	2	3	3	0	47	63	1	0	1	0	0	0	1	1	0
OAB26	22.83	26	5	2	2	1	0	39	47	0	0	1	0	0	0	0	0	0
OAB27	18.37	25	8	1	2	5	0	69	45	0	0	1	0	0	0	0	0	0

OAB28	17.78	25	4	1	1	2	0	45	43	1	1	1	0	0	0	0	0	0
OAB29	20.31	24	10	2	3	5	0	47	55	0	0	1	0	0	0	0	0	0
OAB30	19.38	24	6	1	2	3	0	46	47	1	1	1	0	0	0	0	0	0
C1	23.83	65	2	1	1	0	0	49	50	1	1	0	0	0	0	0	0	0
C2	19.63	59	2	1	0	1	0	34	44	1	1	0	0	0	0	0	0	0
C3	23.88	54	3	0	1	1	1	53	44	1	1	0	0	0	0	0	0	0
C4	20.31	53	0	0	0	0	0	30	34	1	1	0	0	0	0	0	1	0
C5	25.08	50	3	1	2	0	0	44	38	1	1	0	0	0	0	0	0	0
C6	20.03	50	0	0	0	0	0	25	25	1	1	1	0	0	0	0	0	0
C7	23.88	45	1	1	0	0	0	40	34	1	1	1	0	0	0	0	0	0
C8	27.77	44	1	0	1	0	0	61	48	1	1	1	0	0	0	0	1	1
C9	20.43	40	6	2	2	1	1	48	53	1	1	1	0	0	0	0	0	0
C10	19.77	27	4	0	2	2	0	55	60	1	1	1	0	0	0	0	0	0
C11	18.83	27	0	0	0	0	0	44	43	1	1	1	0	0	0	0	0	0
C12	17.48	26	3	0	2	1	0	40	45	1	0	1	0	0	0	0	0	0
C13	19.53	26	0	0	0	0	0	39	41	1	0	1	0	0	0	0	1	0
C14	22.83	26	1	0	0	1	0	39	38	1	0	1	0	0	0	0	0	0
C15	20.81	25	4	1	2	1	2	61	48	1	1	1	0	0	0	0	0	0
C16	20.31	24	0	0	0	0	0	53	35	0	0	1	0	0	0	0	0	0
C17	19.78	24	0	0	0	0	0	31	31	1	1	1	0	0	0	0	0	0
C18	17.31	24	0	0	0	0	0	26	28	1	0	1	0	0	0	0	0	0
C19	19.72	23	1	1	0	0	0	41	46	1	0	1	0	0	0	0	0	0
C20	20.08	23	0	0	0	0	0	38	44	1	1	1	0	0	0	0	0	0
C21	18.07	23	0	0	0	0	0	45	40	0	0	1	0	0	0	0	0	0
C22	20.08	23	0	0	0	0	0	29	28	0	0	1	0	0	0	0	0	0
C23	18.67	22	1	1	0	0	0	45	44	0	0	1	0	0	0	0	0	0
C24	18.37	21	0	0	0	0	0	30	29	0	0	1	0	0	0	0	1	0
C25	19.20	21	0	0	0	0	0	25	25	0	0	1	0	0	0	0	0	0

For binary variables, "1" means "yes"; "2" means "no".
