# Supplementary material for: Urinary Microbiome and Psychological Factors in Women with Overactive Bladder
Source: Front Cell Infect Microbiol. 2017 Nov 27;7:488. doi: 10.3389/fcimb.2017.00488 (PMC5712163; doi:10.3389/fcimb.2017.00488)
Supplement: Supplementary file 2 [file Table2.pdf]

Supplementary table 2- The indices of bacterial alpha diversity in all samples

ID	Chao1	Observed Speci	Shannon	Simpson	Pielous
OAB1	1532.32	748	3.74	0.68	0.47
OAB2	1305.3	959	4.4	0.72	0.38
OAB3	795.68	550	3.81	0.77	0.38
OAB4	1225	504	3.84	0.86	0.36
OAB5	515.33	329	1.76	0.36	0.76
OAB6	592.25	388	3.81	0.83	0.74
OAB7	1298.33	770	6.04	0.94	0.51
OAB8	667.28	291	1.76	0.43	0.03
OAB9	2532.05	624	2.83	0.62	0.34
OAB10	4646.11	1328	5.72	0.94	0.14
OAB11	1911.42	1024	5.05	0.87	0.61
OAB12	290.1	101	0.6	0.14	0.01
OAB13	3483.88	1061	5.07	0.89	0.27
OAB14	2867.34	926	4.88	0.92	0.29
OAB15	4250.45	1094	5.05	0.89	0.26
OAB16	1130	415	1.58	0.33	0.47
OAB17	1920.92	870	5.15	0.9	0.62
OAB18	513.28	243	1.53	0.44	0.13
OAB19	3665.32	1184	5.22	0.92	0.01
OAB20	3669.6	1205	5.36	0.92	0.22
OAB21	1287	577	3.91	0.79	0.17
OAB22	1817.01	657	4.36	0.89	0.64
OAB23	2248.03	1515	6.5	0.92	0.70
OAB24	1976.03	821	4.76	0.89	0.15
OAB25	5056.78	1751	5.5	0.89	0.32
OAB26	2383.37	1618	5.71	0.79	0.25
OAB27	374.83	226	1.61	0.34	0.02
OAB28	1713.09	1113	4.58	0.73	0.40
OAB29	1064.34	678	5.84	0.89	0.58
OAB30	1373.94	727	4.81	0.85	0.34
C1	652.7	319	3.18	0.75	0.60
C2	4945.9	1983	6.44	0.95	0.26
C3	2429.67	934	3.5	0.75	0.52
C4	1794.45	1338	8.13	0.97	0.31
C5	1719.16	655	2.74	0.57	0.58
C6	1365.14	730	4.73	0.91	0.32
C7	3573.66	1446	5.83	0.93	0.31
C8	3635.21	809	2.87	0.6	0.17
C9	1603.96	1049	4.18	0.69	0.73
C10	2011.44	743	4.8	0.89	0.45
C11	1608.89	570	3.76	0.81	0.44
C12	5586.45	1982	6.84	0.97	0.33
C13	4529.99	1374	5.57	0.93	0.75

C14	6075.53	2182	6.9	0.97	0.33
C15	2946.63	1120	5.95	0.96	0.54
C16	660.53	362	2.8	0.71	0.27
C17	1674.29	632	4.33	0.85	0.59
C18	1086.41	541	3.7	0.82	0.20
C19	6817.21	2384	7.17	0.98	0.38
C20	1340.22	614	3.77	0.82	0.17
C21	3425.63	915	4.56	0.86	0.76
C22	3837.43	2823	9.19	0.98	0.46
C23	2026.46	800	4.77	0.88	0.51
C24	2092.89	762	4.49	0.9	0.25
C25	2810.04	2038	9.05	0.99	0.38
