# Supplementary material for: Urinary Microbiome and Psychological Factors in Women with Overactive Bladder
Source: Front Cell Infect Microbiol. 2017 Nov 27;7:488. doi: 10.3389/fcimb.2017.00488 (PMC5712163; doi:10.3389/fcimb.2017.00488)
Supplement: Supplementary file 3 [file Table3.pdf]

Supplementary table 3 - Differences between these two OAB subgroups based on hierarchical clustering of weighted UniFrac distance metric

Variables	OAB patients in cluster 1 and cluster 3 (n=16)	OAB patients in cluster 2 (n=14)	*P* value
BMI	20.70	20.24	0.56
Age (y)	35.44	29.36	0.14
OABSS	8.00	7.50	0.54
Daytime frequency	1.75	1.57	0.32
Nighttime frequency	2.06	2.21	0.58
Urgency	3.75	3.50	0.61
Urgency incontinence	0.56	0.21	0.40
SDS	53.61	49.54	0.21
SAS	52.59	50.58	0.52
Chao1	1921.74	1954.18	0.95
Observed Species	845.94	768.71	0.63
Shannon	4.61	3.64	0.10
Simpson	0.66	0.82	0.07
Pielous	0.41	0.28	0.10
