# Supplementary material for: Urinary Microbiome and Psychological Factors in Women with Overactive Bladder
Source: Front Cell Infect Microbiol. 2017 Nov 27;7:488. doi: 10.3389/fcimb.2017.00488 (PMC5712163; doi:10.3389/fcimb.2017.00488)
Supplement: Supplementary file 5 [file Image1.PDF]

A

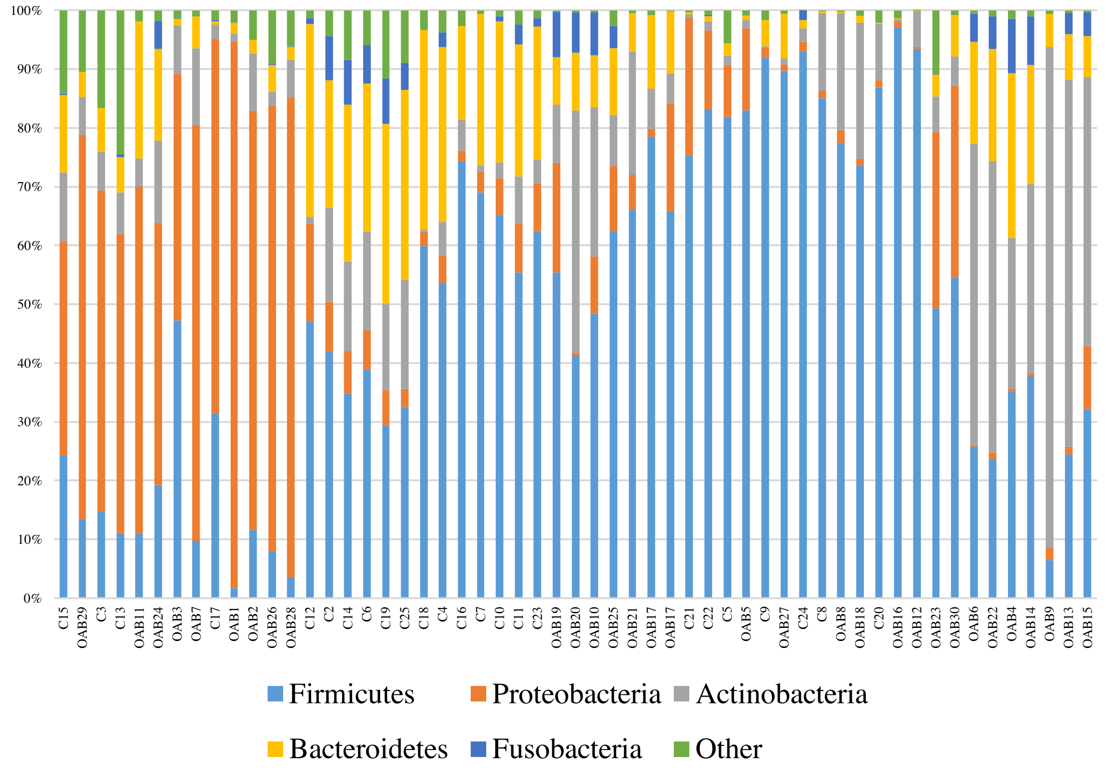
100%
90%
80%
70%
60%
50%
40%
30%
20%
10%
0%
C15 OAB29 C3 C13 OAB11 OAB24 OAB3 OAB7 C17 OAB1 OAB2 OAB26 OAB28 C12 C2 C14 C6 C19 C25 C18 C4 C16 C7 C10 C11 C23 OAB19 OAB20 OAB10 OAB25 OAB21 OAB17 OAB17 C21 C22 C5 OAB5 C9 OAB27 C24 C8 OAB8 OAB18 C20 OAB16 OAB12 OAB23 OAB30 OAB6 OAB22 OAB4 OAB14 OAB9 OAB13 OAB15
Firmicutes
Proteobacteria
Actinobacteria
Bacteroidetes
Fusobacteria
Other

B

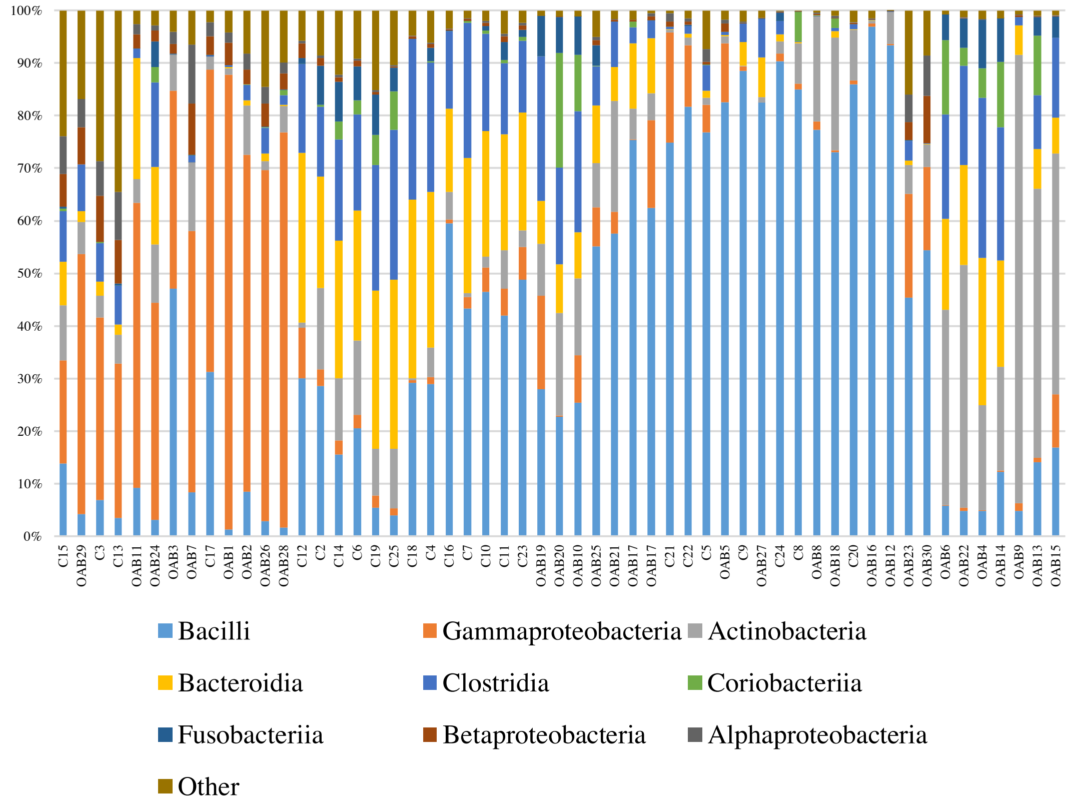
100%
90%
80%
70%
60%
50%
40%
30%
20%
10%
0%
C15 OAB29 C3 C13 OAB11 OAB24 OAB3 OAB7 C17 OAB1 OAB2 OAB26 OAB28 C12 C2 C14 C6 C19 C25 C18 C4 C16 C7 C10 C11 C23 OAB19 OAB20 OAB10 OAB25 OAB21 OAB17 OAB17 C21 C22 C5 OAB5 C9 OAB27 C24 C8 OAB8 OAB18 C20 OAB16 OAB12 OAB23 OAB30 OAB6 OAB22 OAB4 OAB14 OAB9 OAB13 OAB15
Bacilli
Gammaproteobacteria
Actinobacteria
Bacteroidia
Clostridia
Coriobacteriia
Fusobacteriia
Betaproteobacteria
Alphaproteobacteria
Other

C

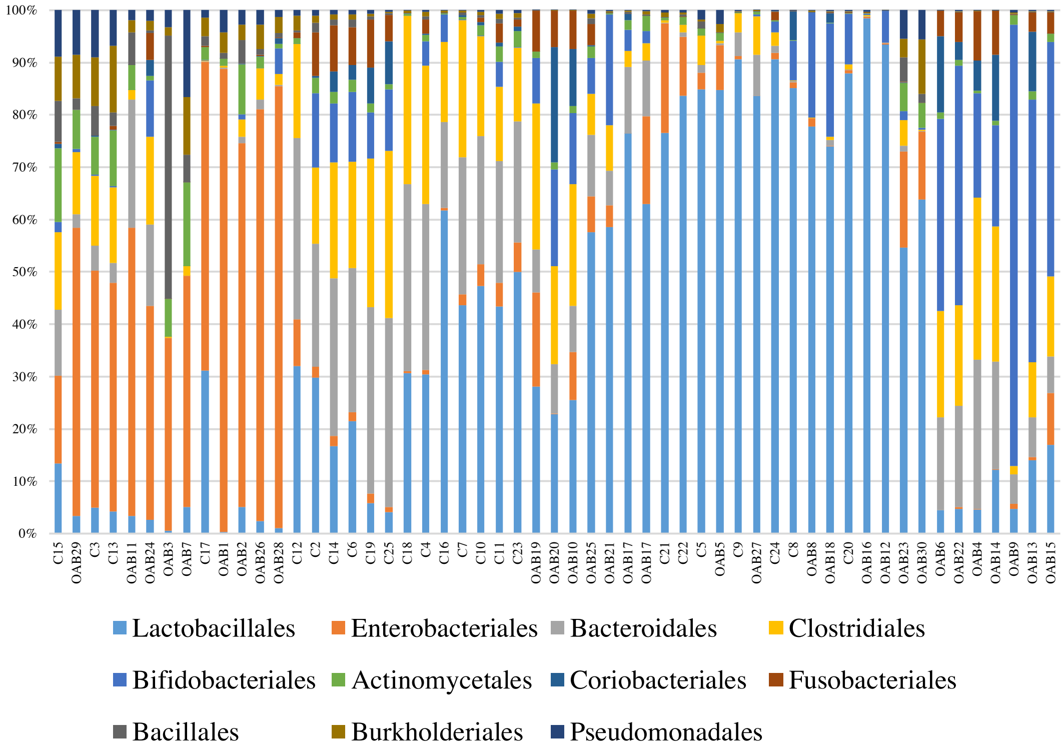

100%
90%
80%
70%
60%
50%
40%
30%
20%
10%
0%
C15 OAB29 C3 C13 OAB11 OAB24 OAB3 OAB7 C17 OAB1 OAB2 OAB26 OAB28 C12 C2 C14 C6 C19 C25 C18 C4 C16 C7 C10 C11 C23 OAB19 OAB20 OAB10 OAB25 OAB21 OAB17 OAB17 C21 C22 C5 OAB5 C9 OAB27 C24 C8 OAB8 OAB18 C20 OAB16 OAB12 OAB23 OAB30 OAB6 OAB22 OAB4 OAB14 OAB9 OAB13 OAB15
Lactobacillales
Enterobacteriales
Bacteroidales
Clostridiales
Bifidobacteriales
Actinomycetales
Coriobacteriales
Fusobacteriales
Bacillales
Burkholderiales
Pseudomonadales

D

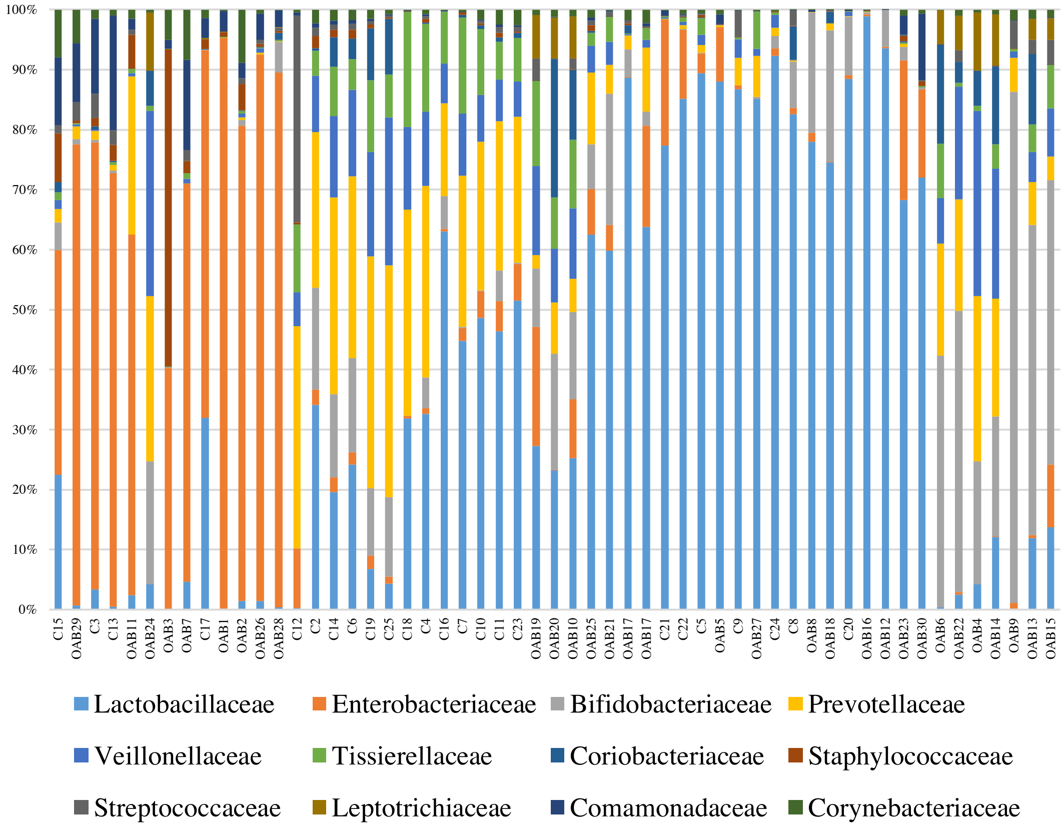

100%
90%
80%
70%
60%
50%
40%
30%
20%
10%
0%
C15 OAB29 C3 C13 OAB11 OAB24 OAB3 OAB7 C17 OAB1 OAB2 OAB26 OAB28 C12 C2 C14 C6 C19 C25 C18 C4 C16 C7 C10 C11 C23 OAB19 OAB20 OAB10 OAB25 OAB21 OAB17 OAB17 C21 C22 C5 OAB5 C9 OAB27 C24 C8 OAB8 OAB18 C20 OAB16 OAB12 OAB23 OAB30 OAB6 OAB22 OAB4 OAB14 OAB9 OAB13 OAB15
Lactobacillaceae
Enterobacteriaceae
Bifidobacteriaceae
Prevotellaceae
Veillonellaceae
Tissierellaceae
Coriobacteriaceae
Staphylococcaceae
Streptococcaceae
Leptotrichiaceae
Comamonadaceae
Corynebacteriaceae

E

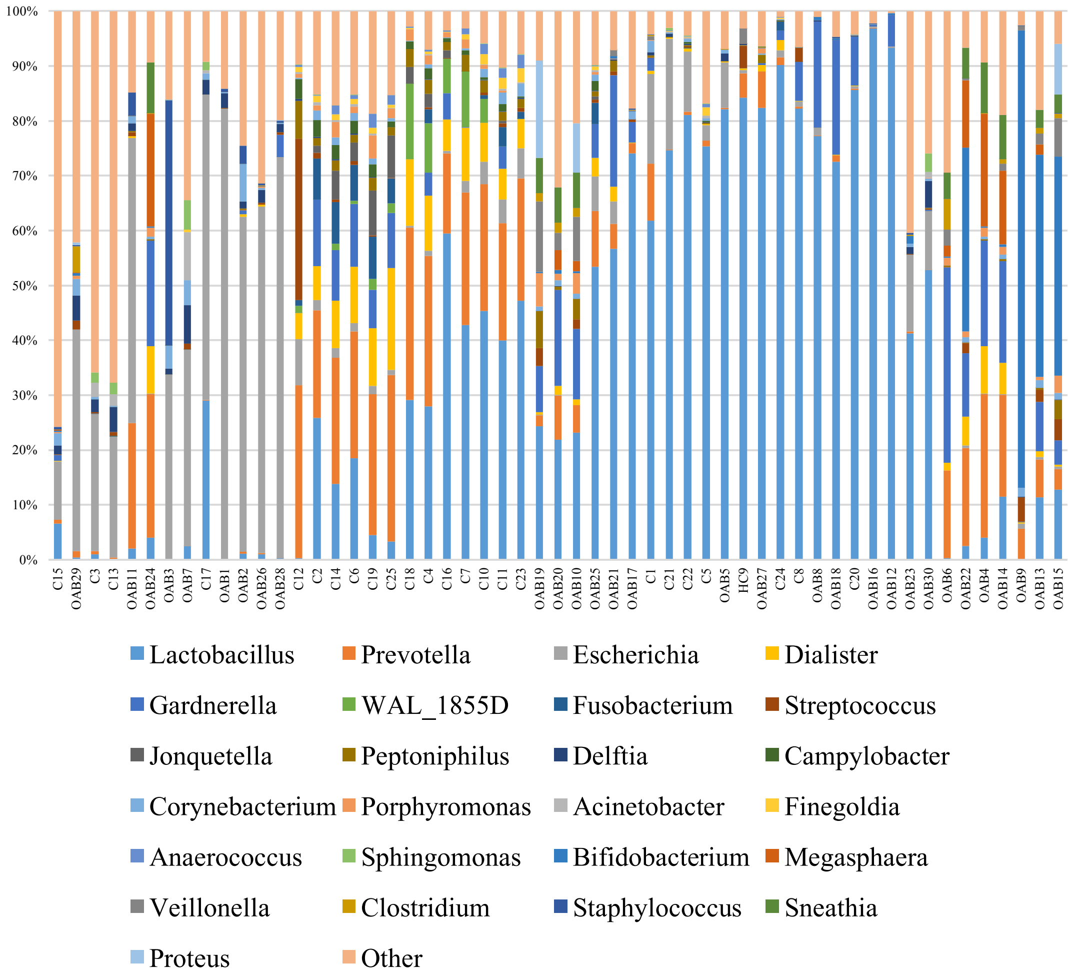

Supplementary figure 1. Bar charts of different classification levels in urinary microbiota of OAB patients and healthy controls.

(A) phylum; (B) Class; (C)Other; (D)family; (E)genus.
